# The Landscape of Small Non-Coding RNAs in Triple-Negative Breast Cancer

**DOI:** 10.3390/genes9010029

**Published:** 2018-01-10

**Authors:** Yan Guo, Hui Yu, Jing Wang, Quanhu Sheng, Shilin Zhao, Ying-Yong Zhao, Brian D. Lehmann

**Affiliations:** 1 Department of Internal Medicine, University of New Mexico, Albuquerque, NM 87131, USA; yaguo@salud.unm.edu (Y.G.); huiyu1@salud.unm.edu (H.Y.); 2 Key Laboratory of Resource Biology and Biotechnology in Western China, School of Life Sciences, Northwest University, Xi’an 710069, Shaanxi, China; zyy@nwu.edu.cn; 3 Department of Biostatistics, Vanderbilt University Medical Center, Nashville, TN 37232, USA; jing.wang.1@vanderbilt.edu (J.W.); quanhu.sheng.1@vanderbilt.edu (Q.S.); Shilin.zhao.1@vanderbilt.edu (S.Z.)

**Keywords:** small RNA, miRNA, TNBCtype, biomarker

## Abstract

Triple-negative breast cancer (TNBC) is an operational term for breast cancers lacking targetable estrogen receptor expression and HER2 amplifications. TNBC is, therefore, inherently heterogeneous, and is associated with worse prognosis, greater rates of metastasis, and earlier onset. TNBC displays mutational and transcriptional diversity, and distinct mRNA transcriptional subtypes exhibiting unique biology. High-throughput sequencing has extended cancer research far beyond protein coding regions that include non-coding small RNAs, such as miRNA, isomiR, tRNA, snoRNAs, snRNA, yRNA, 7SL, and 7SK. In this study, we performed small RNA profiling of 26 TNBC cell lines, and compared the abundance of non-coding RNAs among the transcriptional subtypes of triple negative breast cancer. We also examined their co-expression pattern with corresponding mRNAs. This study provides a detailed description of small RNA expression in triple-negative breast cancer cell lines that can aid in the development of future biomarker and novel targeted therapies.

## 1. Introduction

Triple-negative breast cancer (TNBC) is an aggressive form of breast cancer, representing ~15% of cases, but ~25% of all breast cancer deaths. TNBCs are defined by their absence of estrogen and progesterone receptor expression and HER2 amplifications. Lacking these biomarkers, TNBCs are insensitive to current targeted therapies. Molecular heterogeneity, and a lack of high-frequency “driver” alterations amenable to therapeutic intervention have hindered the development of new treatments for TNBC [1]. We have previously classified TNBC into distinct transcriptional subtypes, each with unique biology and signaling [2]. Four tumor intrinsic subtypes include basal-like (BL1, BL2), mesenchymal (M), and luminal androgen receptor (LAR), each of which can be additionally classified by an immunomodulatory (IM) descriptor on the basis of immune infiltrate presence [3]. These transcriptional subtypes have been independently validated by several other groups [4,5]. The initial subtyping studies were performed on microarray gene expression, however, high-throughput sequencing (HTS) has since evolved as an unbiased method to quantify the expression of many RNA species outside of coding transcripts.

Small RNAs (sRNAs) are short non-coding RNAs (ncRNA) of 200 nucleotides or less in length. The discovery of novel sRNAs and their annotations have substantially enhanced our understanding of the complex regulation of the genome. MicroRNAs (miRNAs) are, by far, the most studied small RNA, with over 10,000 publications to date. Discovered in 1993 [6], miRNAs are single-stranded ncRNAs of 19–25 nucleotides that regulate messenger RNAs (mRNAs) through binding of the seed sequence (first 2 to 7 nucleotides) to complementary bases in 3′ untranslated regions (3’UTR) of mRNA. miRNAs have been recognized for their potential to serve as biomarkers for various diseases, including cancer [7,8,9,10,11,12]. The relatively high transcript number, stable biochemical properties under clinical conditions, and discriminating transcriptional patterns make miRNAs ideal candidates for biomarkers.

Earlier studies of sRNA have relied on low-throughput real-time quantitative polymerase chain reaction (RT-PCR) or hybridization-based microarrays. The advancement of HTS technology has substantially increased the detection limit of miRNAs, and more importantly, enabled the examination of miRNA at a single nucleotide resolution, in addition to quantifying abundance. Furthermore, HTS enables a global assessment of sRNAs and not limited to a set of previously known targets. Subsequent bioinformatics analysis of sRNA sequencing data can identify, quantify and determine the differential expression of a variety of small non-coding RNAs. Since size-selection sequencing methods are agnostic to sRNA species, it has the potential to capture many species of sRNAs that include miRNAs, miRNA isoforms (isomiRs) [13,14], transfer RNA (tRNA)-derived small RNAs (tDRs) [15,16], and other sRNAs such as snRNA, snoRNA, yRNA, 7SK, and 7SL RNAs [17,18]. IsomiRs are the isoforms of miRNA that usually have alternative or clipped seed sequences compared to reference miRNA sequences [19]. The differences in seed sequence of isomiRs can result in substantial difference in the repertoire of predicted target mRNAs [20]. In addition, transfer RNAs (tRNAs) can be detected by HTS, usually as fragments that were actively cleaved or a product of library construction. The parent tRNAs are adaptor molecules with a length typically ranging from 73 to 94 nucleotides. It is speculated that the cleavage of tRNAs by an RNAse III enzyme, angiogenin, may occur in a number of reactive conditions, to produce tRNA-derived halves (tRHs) [21,22].

The present study provides an in-depth characterization of small non-coding RNA species in TNBC, and identifies subtype-specific differences in major non-coding RNA species detectable: micro RNA (miRNA), miRNA isoforms, transfer RNA (tRNA), small nucleolar RNA (snoRNA), small nuclear RNA (snRNA), Y RNA (yRNA), single recognition particle RNA (7SL RNA), and 7SK RNA. These data validate previously identified pathways, and highlight potential biomarkers for future studies.

## 2. Methods

### 2.1. High-Throughput RNA Sequencing

We cultured 26 TNBC cell lines (BT20, BT549, CAL120, CAL148, CAL51, DU4475, HCC1143, HCC1187, HCC1395, HCC1599, HCC1806, HCC1937, HCC38, HCC70, HDQP1, HS578T, MDAMB157, MDAMB231, MDAMB436, MDAMB453, MDAMB468, MFM223, SUM159, SUM185, SW527) for this study; the cell culture procedures were previously described [2]. Sub-confluent cells (1–2 × 10^6^) were harvested and sRNA isolated (mirVana, Thermo Fisher, Waltham, MA, USA) using a standard spin protocol. RNA quality assessment and RNA-seq was performed by the Vanderbilt Technologies for Advanced Genomics core (VANTAGE, Nashville, TN, USA). Libraries were prepared using the TruSeq Small RNA sample preparation kit (Illumina, San Diego, CA, USA). The sRNA protocol specifically ligates RNA adapters to mature miRNAs harboring a 5′-phosphate and 3′-hydroxyl group as a result of enzymatic cleavage by RNase III processing enzymes, e.g., Dicer. In the first step, RNA adapters were ligated onto each end of the sRNA, and reverse transcription was used to create single-stranded cDNA. This cDNA was then PCR amplified for 18 cycles with a universal primer, and a second primer containing one of 20 uniquely indexed tags to allow multiplexing. Size-selection of the cDNA constructs was performed using a 3% gel cassette on the Pippin Prep (Sage Sciences, Beverly, MA, USA) to include only mature miRNAs and other sRNAs in the 5–40 bp size range, and to remove adapter–adapter products. The resulting cDNA libraries then underwent a quality check on the Agilent Bioanalyzer HS DNA assay (Agilent, Santa Clara, CA, USA) to confirm the final library size, and on the Agilent Mx3005P quantitative PCR machine, using the KAPA library quantification kit (Illumina, San Diego, CA, USA) to determine concentration. A 2 nM stock was created, and samples were pooled by molarity for equimolar multiplexing. From the pool, 10 pM of the pool was loaded into each well of the flow cell on the Illumina cBot for cluster generation. The flow cell was then loaded and sequenced on the Illumina HiSeq 3000 to obtain at least 15 million single end (1 × 50 bp) reads per sample. The raw sequencing reads in BCL format were processed through CASAVA-1.8.2 for FASTQ conversion and de-multiplexing. The chastity filter from Illumina’s Real-Time Analysis software was applied, and only PF (pass filter) reads were retained for further analysis. Raw data files available at Gene Expression Omnibus under the accession GSE108286.

We also performed total RNA-seq on the 26 TNBC cell line for comparative purpose. Total RNA was isolated with the Aurum Total RNA Mini Kit (Bio-Rad, Hercules, CA, USA). All samples were quantified on the QuBit RNA assay (Thermo Fisher, Waltham, MA, USA). RNA quality was checked using Agilent Bioanalyzer. RNA integrity number (RIN) for both sRNA and total RNA was 10. The ribosome RNA reduction was performed using the Ribo-Zero Magnetic Gold Kit (Human/Mouse/Rat) (Epicentre, Madison, WI, USA). The RNA libraries were sequenced on Illumina High HiSeq 3000 with paired-end 100 base pair long reads.

### 2.2. Bioinformatics and Data Analyses

HTS data processing was performed using a custom in-house data analysis pipeline Tiger [15] for sRNA sequencing data processing. Cutadapt [23] was used to trim 3′ adapters for raw reads. Multi-perspective quality control [24] on raw data was performed using QC3 [25]. All reads with length less than 16 nucleotides were discarded. The adaptor-trimmed reads were formatted into a non-redundant FASTQ file, where the read sequence and copy number was recorded for each unique tag. The usable unique reads were mapped to the whole genome by Bowtie1 [26], allowing only one mismatch per read. In addition, our pipeline takes into consideration non-templated nucleotide additions [27,28,29,30] at the 3′ end of miRNAs during alignment, resulting in more accurate miRNA expression quantification. The miRNA coordinates were extracted from miRBase [31]. The tRNA coordinates were prepared by combining the latest UCSC tRNA database GtRNAdb [32] with the tRNA loci of mitochondria from the Ensembl database [33]. The coordinates of snRNA, snoRNA, yRNA, 7SK, and 7SL were extracted from the Ensembl database. The tRNA reads were used not only for tRNA quantification, but also for tRNA mapping position coverage analysis. sRNAs were divided into seven major categories: miRNAs (including isomiR), tRNAs, 7SK, 7SL, yRNA, snoRNAs, and snRNAs. IsomiRs were detected by matching alignment of the reads at +1 or +2 positions from the start of the 5′ annotation of miRNAs.

The total RNA-seq data was quality controlled following standard RNA-seq quality control protocols [24] using tools QC3 [25], and alignment was carried out using BWA [34]. The nuclear genome we used is the human reference genome GRCh38. RNA data alignment was performed by TopHat2 [35]. Read count per gene was extracted using HTSeq [36]. For total RNA-seq, we sequenced, on average, 34 million reads per sample.

Differential sRNA expression analysis among subtypes of TNBC cell lines were performed by MultiRankSeq (Nashville, TN, USA) [37]. Due to limitation on the current curation on sRNA functions, pathway analysis was only performed with miRNA. Correlation based co-expression network analysis were performed with Cytoscape (San Francisco, CA, USA). Differentially expressed miRNAs pathway analysis was performed using miRPath v3 [38]. All correlation analyses were performed with Spearman’s correlation to minimize the effect of outliers. The Cancer Genome Atlas (TCGA) miRNA and mRNA data for TNBC tumors were downloaded from Genomic Data Commons (https://portal.gdc.cancer.gov/) to validate the correlation patterns identified in this study.

### 2.3. TNBC Subtyping

We performed TNBC subtyping as previously described [2,39], using RSEM gene-level expression estimates determined from RNA-sequencing of each cell line. Cell lines were assigned one of four tumor-intrinsic TNBC subtypes: basal-like 1 (BL1), basal-like 2 (BL2), mesenchymal (M), and luminal androgen receptor (LAR) [39].

## 3. Results

**Figure 1:**
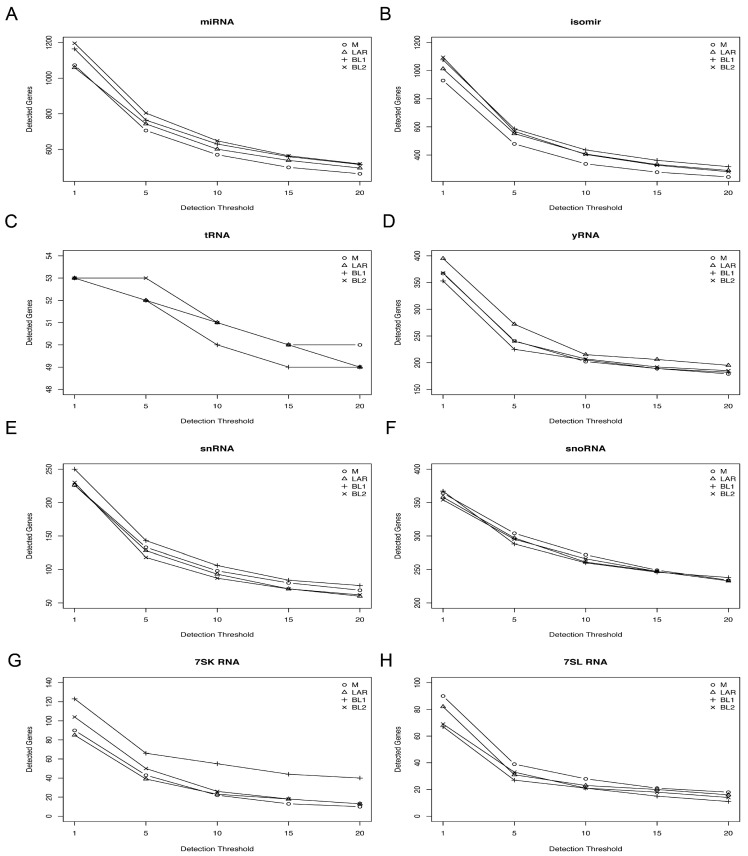
Number of detected sRNA by detection thresholds of read count. Plot shows number of sRNAs identified at incremental detection threshold (read counts) (1, 5, 10, 15, 20). The number of sRNA detected should decrease as the detection threshold increases. (**A**) miRNA, (**B**) isomer, (**C**) tRNA, (**D**) yRNA, (**E**) snRNA, (**F**) snoRNA, (**G**) 7SK and (**H**) 7SL RNA species with each triple-negative breast cancer (TNBC) subtype.

**Figure 2:**
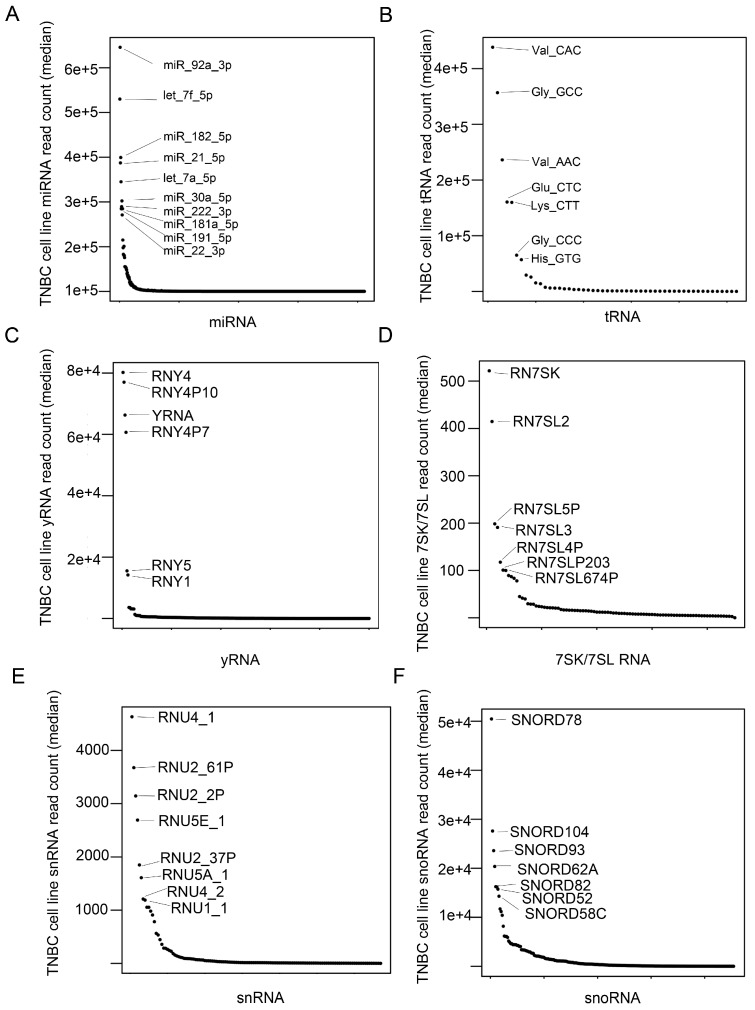
Highly expressed non-coding RNAs in TNBC cell lines. Scatterplots show median read counts for (**A**) miRNA, (**B**) tRNA, (**C**) yRNA, (**D**) 7SK/7SL RNA, (**E**) snRNA and (**F**) snoRNA across all TNBC cell lines.

To determine if different species of small RNAs species are enriched in TNBC subtypes, we performed sRNA sequencing of 26 TNBC cell lines (Table S1). After quality control, we generated a sRNA dataset that identified 823 miRNAs, 3762 isomiRs, 52 tRNAs, 134 snRNAs, 305 snoRNAs, 250 yRNAs, 57 7SK RNAs, and 33 7SL RNAs (Table S2). We observed a slight sRNA preference by TNBC subtype. For example, for cell lines of mesenchymal subtype, we identified more miRNA than other subtypes, and basal-like 1 (BL1) cell lines expressed higher levels of snRNA and 7SK RNAs (Figure 1). To determine if sRNA species are associated with TNBC transcriptional subtypes, we performed unsupervised hierarchical clustering of miRNA, tRNA, snRNA, snoRNA, yRNA, and 7SL/7SK RNA species (Figure S1). To identify small non-coding RNAs highly expressed in TNBC, we plotted the median expression across all cell lines by RNA species (Figure 2). Several miRNAs (miR-92a-3p, let-7f-5p, miR-182-5p, miR-21-5p, let-7a-5p, miR-30a-5p, miR-222-3p, miR-181s-5p, miR-191-5p, miR-22-3p) were highly expressed among the cell lines (Figure 2A). There were several tRNA species highly expressed (Val_CAC, Gly_GCC, Val_ACC, Glu_CTC, Lys_CTT, Gly_CCC, and His_GTG) across the cell lines (Figure 2B), as well as yRNAs, 7SK/7SL RNAs, snRNAs, and snoRNAs (Figure 2C,D).

**Figure 3:**
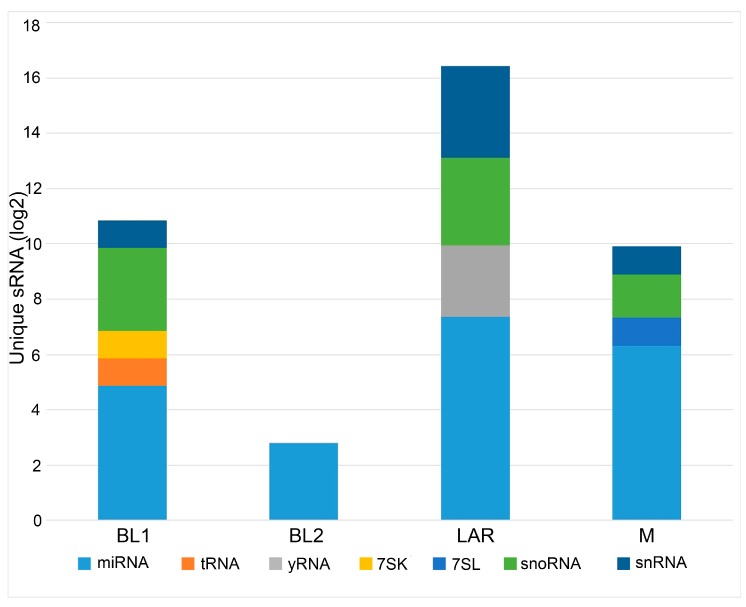
Differential enrichment of sRNAs by TNBC subtype. Barplot shows relative number of differential sRNAs colored by species type and binned by TNBC subtypes.

To identify uniquely enriched non-coding RNAs, we conducted differential expression analysis between each of the TNBC subtypes (Table 1). Additionally, differential expression analysis was performed to identify unique sRNAs pertaining to each TNBC subtype compared to all other TNBCs (Table 2). Not surprisingly the LAR was found to be the most unique TNBC subtype with 104 exclusive sRNAs, supporting the distinct hormonally driven biology of this subtype. BL2 was the least unique TNBC subtype with six exclusive sRNAs. BL1 and M had 32 and 48 exclusive sRNAs respectively (Figure 3). The complete lists of exclusive sRNAs pertaining to each TNBC subtype is listed in Table S3.

There were differences in the expression of small nuclear RNA (snRNA) components of spliceosomes, transcription elongation, and signal recognition particle complexes across TNBC subtypes. Spliceosomes are integral to eukaryotic precursor messenger RNA maturation. BL1 subtype cell lines were enriched for U7 small nuclear RNAs that are involved in histone pre-mRNA processing (RNU7-19P and RNU7-3P), while the LAR subtype displayed lower levels of U2 snRNAs (RNU2-33P, RNU2-36P, RNU2-37P, RNU2-48P, RNU2-50P, RNU2-61P, and RNU2-7P). The BL1 subtype was enriched in the 7SK snRNA component of the positive transcription elongation factor P-TEFb [40]. The secondary structure of 7SK associates with several proteins, regulating the stability and activity of the ribonucleoprotein complex. The M subtype displayed enrichment in the 7SL snRNA, the RNA component of the signal recognition particle ribonucleoprotein complex. This universally conserved ribonucleoprotein processes the signal peptide present on proteins destined for secretion. Several H/ACA family snoRNAs were differentially enriched in the BL1 subtype that include SNORA11, SNORA21, SNORA48, SNORA68, and SNORA74B that can guide 2′-*O*-methylation of target RNA. yRNAs are components of the autoantigenic Ro ribonucleoproteins were uniquely enriched in the LAR subtype. yRNAs are overexpressed in various cancers, [41] and implicated in chromosomal DNA replication and non-coding RNA quality control [42,43].

**Figure 4:**
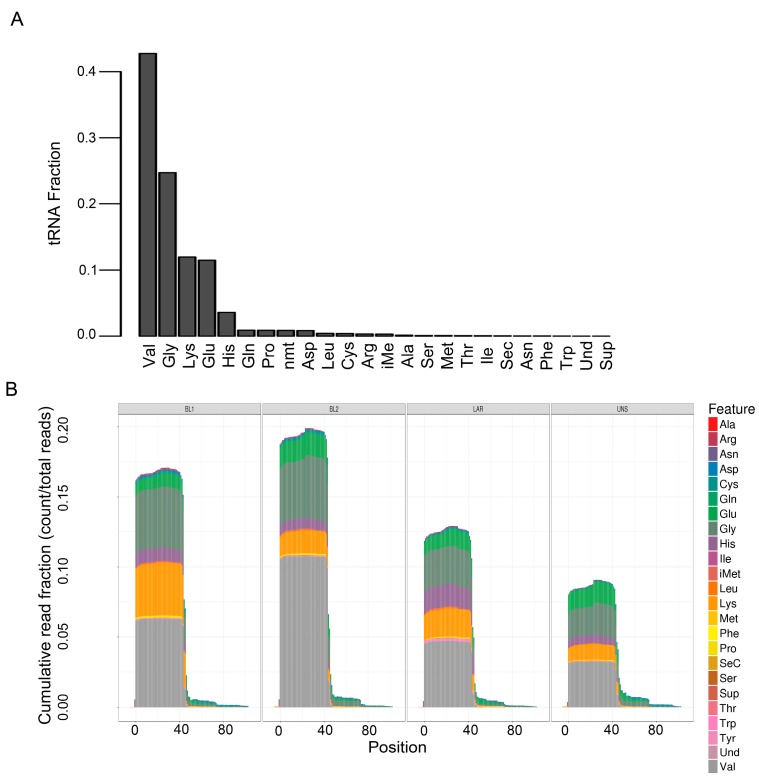
Quantification and differential enrichment of tRNA species across TNBC subtypes. (**A**) Barplot shows average fraction of tRNA species across all TNBC cell lines. (**B**) Plot shows tRNA quantification by nucleotide position for all tRNA species in cell lines grouped by TNBC subtype. The sequenced tRNA fragments overwhelmingly favor the first half (~0–40 positions) of tRNA.

Transfer RNAs (tRNAs) are adaptor molecules, typically 76 to 90 nucleotides in length that physically link mRNA and the amino acid sequence of proteins. tRNAs deliver individual amino acids to ribosomes for protein translation. Val, Gly, Lys, Glu, and His were by far the most abundant tRNAs across all TNBC cell lines, representing ~95% of all tRNAs (Figure 4A). tRNA differential expression analysis was performed based on the anti-codon categories. The only significant difference in tRNA species among TNBC subtypes were BL1 cell lines that displayed significantly less tRNA-Ser (0.07%) compared to other subtypes (0.16% BL2, 0.12% LAR and 0.17% M). The rest tRNA anti-codon categories were represented equally in proportion for all TNBC subtypes (Figure 4B).

Since miRNA were the most abundant non-coding RNA species and have the ability to regulate diverse pathways by targeting mRNAs, we performed pathway analysis on miRNAs exclusive to each TNBC subtype (Table S4). As anticipated, the M subtype was enriched in miRNAs regulating mesenchymal pathways, such as Wnt signaling, TGF beta signaling, adherens junction, and axon guidance. Specifically, the miRNA-200 cluster targeting transcription factors that regulate E-cadherin were decreased in the M subtype, supporting prior published enrichment in epithelial-to-mesenchymal (EMT) genes [2].

**Figure 5:**
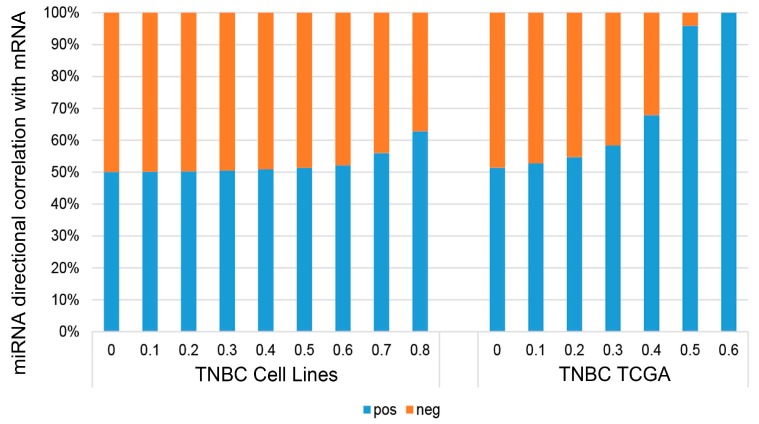
Directional correlation of miRNAs to mRNA expression in TNBC cell lines and TNBC tumors in TCGA. Barplots show the relative fractions of miRNAs that correlate either in the positive or negative direction with mRNAs binned by increasing correlation strength in TNBC cell lines and TNBC tumors in TCGA.

We performed correlation analyses between the expression of sRNA and mRNA in TNBC cell lines. All correlations were computed using Spearman’s correlation to minimize outlier and scaling effects between datasets. We found that there is a slight bias toward positive correlation for miRNA vs. mRNA in our TNBC cell lines; this bias increased with higher absolute correlation values (Figure 5). To ensure this observation is not an artifact of our cell line dataset, we performed the same analysis using gene expression data from primary TNBC tumor in The Cancer Genome Atlas (TCGA), and observed the same bias toward positive correlation. Two additional studies [44,45] also found similar positive bias for correlations between miRNA and mRNA. The plausible explanation is that miRNAs also target inhibitive transcription factors of other mRNAs [46].

**Figure 6:**
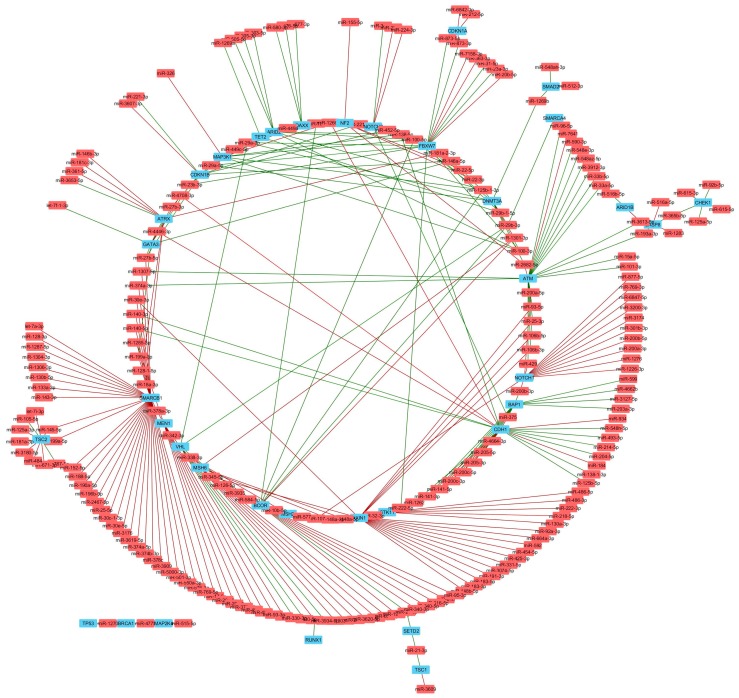
Tumor suppressor genes significantly correlated with miRNAs in TNBC. Network analysis shows miRNAs (red) that negatively (red lines) or positively (green lines) correlate with mRNA expression, or genes encoding known tumor suppressor genes (blue).

**Figure 7:**
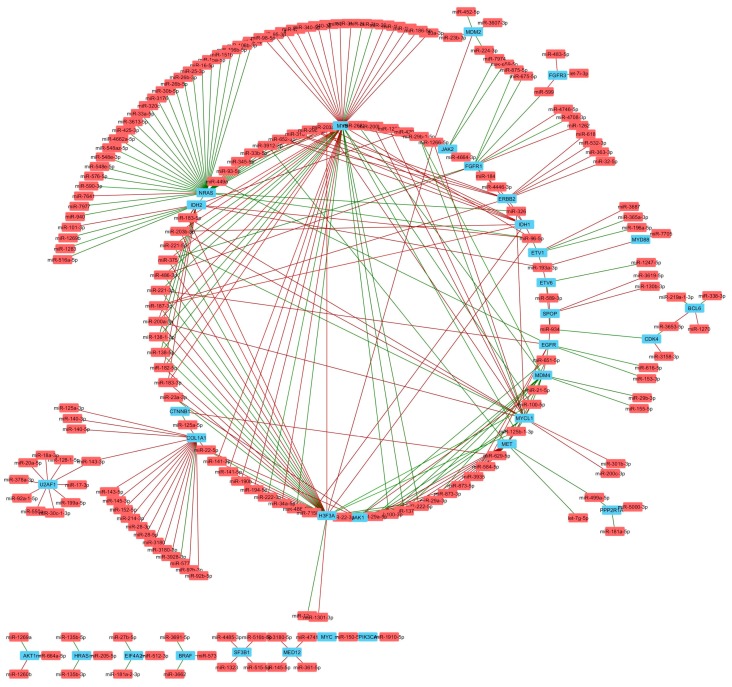
Oncogenes significantly correlated with miRNAs in TNBC. Network analysis shows miRNAs (red) that negatively (red lines) or positively (green lines) correlate with mRNA expression of genes encoding known oncogenes (blue).

**Figure 8:**
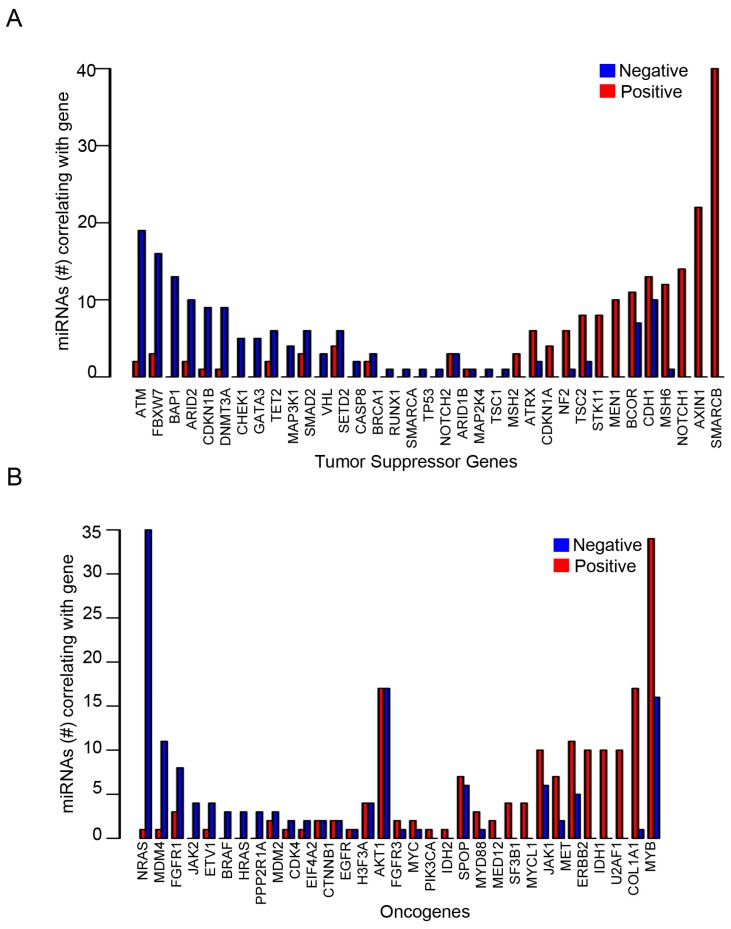
Differential enrichment of miRNAs targeting tumor suppressor and oncogenes in TNBC. Barplots show the number of miRNAs correlating in the negative or positive direction with known (**A**) tumor suppressor genes or (**B**) oncogenes in TNBC cell lines.

To further study the correlations in detail, we constructed a list of known tumor suppressor genes and oncogenes (Table S5). The notable miRNA gene targets altered by TNBC subtype are listed in Table 3. The correlations between miRNA and tumor suppressor genes and oncogenes were selected for co-expression analysis (Figure 6 and Figure 7). Tumor suppressor genes involved in DNA damage repair (ATM, BAP1, CHEK1, and BRCA1), chromatin modifying genes (ARID2, DNMT3A, TET2, SETD2, and GATA3) and cell cycle genes (CDKN1B and FBXW7) were enriched in the number of miRNAs negatively correlated with mRNA expression (Figure 8A). FBXW7 encodes an F-box protein and part of the ubiquitin protein ligase SKP1-cullin-F-box (SCF) complex that negatively regulates cyclin E, c-MYC, and notch1 proteins, of which cyclin E has been shown to be a specific marker in basal-like breast cancer [47]. Furthermore, CDKN1B encodes the cyclin-dependent kinase inhibitor p27 that prevents G1/S cell cycle progression by inhibiting cyclin E/cdk2-dependent Rb phosphorylation. These data implicate miRNAs in direct regulation of Rb activity, which is frequency diminished [48] or lost in basal-like TNBC [49]. Increased miRNAs that correlate with decreased DNA damage repair protein observed in TNBC cell lines may complement other mechanisms of BRCA1 pathway inactivation outside of BRAC1 mutation and promoter hypermethylation. Interestingly, miRNAs that positively correlated with mRNA expression were enriched in growth factor signaling (NF2, TSC2 and STK11) and developmental pathways (NOTCH1 and AXIN1), suggesting that these miRNAs may target a negative regulator of these tumor suppressors (Figure 8A). Oncogenes that negatively correlated with miRNAs were enriched in MAPK pathway (NRAS, BRAF, HRAS and ETV1), while positively correlated genes were enriched in MYC genes (MYC and MYCL1) (Figure 8B).

A summary network of tumor suppressor and oncogenes correlated with all sRNA species is provided in Supplemental Figures S2 and S3. Of note, many yRNA species were negatively correlated with the expression of cell cycle gene CCNE1, and these sRNAs are necessary for DNA replication through interactions with chromatin and initiation proteins [50]. Small nucleolar RNAs guide chemical modifications of other RNAs, and we identified several small nucleolar RNAs positively correlated with the splicing gene SF3B1, including SNORD116, that was identified in a bioinformatics screen to be associated with alternatively spliced genes, suggesting a role in alternative splicing [51].

## 4. Discussion

The functions of non-coding RNAs are continuously being uncovered, and are implicated in epigenetic, transcriptional, and post-transcriptional regulation. NGS has expanded our ability to investigate RNA expression outside of the coding genome. While many studies have implicated miRNAs that associate with prognosis, little is known about the expression pattern of non-coding RNAs in triple-negative breast cancers [52,53]. Using TNBC cell line models, we performed a global analysis of small non-coding RNAs. Using corresponding mRNA expression, we identified tumor suppressor and oncogenes that correlated with miRNA expression. The expression of several tumor suppressor genes was decreased, and correlated with increased miRNA expression. These were enriched in DNA damage, cell cycle checkpoints, and chromatin modifying genes. It is likely that these associated miRNAs may serve to inhibit the function of these specific tumor suppressor pathways in TNBC.

Using differential expression analysis, we identified non-coding RNAs that are associated with biological TNBC subtypes. The majority of the differentially expressed small non-coding RNAs were miRNAs, and correlation with mRNA validated several targets. The BL1 and BL2 subtypes were enriched in mRNA targeting members of the ErbB receptor tyrosine kinase family. However, the BL2 subtype displayed enrichment in miRNAs regulating Wnt signaling and pathways regulating pluripotency of stem cells. Furthermore, the BL2 subtype was enriched in miRNAs regulating calcium and cyclic GMP protein kinase G signaling, suggesting modulation of this pathway made be potentially therapeutic as in head and neck squamous cancer [54]. The LAR subtype was enriched in miRNAs regulating biosynthetic pathways known to be regulated by androgen receptor, such as fatty acid biosynthesis [55], and *N*-glycan biosynthesis [56,57]. As expected, the mesenchymal TNBC subtype is characterized by enrichment in mRNAs regulating adherens junction, axon guidance, TGF-beta, and Wnt signaling. There was enrichment in EMT gene expression and loss of epithelial markers, like E-cadherin, in the M subtype. We confirmed decreased expression of the miRNA-200 cluster and decreased E-cadherin in the mesenchymal subtype. These data support prior studies demonstrating miR200 targets the zinc finger E-box-binding homeobox (ZEB2) transcription factor suppression of E-cadherin transcription [58].

In addition to miRNA, we identified several other species that were differentially enriched in TNBC subtypes. The BL1 subtype cell lines were enriched in U7 snRNAs that are involved in histone pre-mRNA processing, likely due to the increased cell cycle and proliferation associated with this subtype [2]. The BL1 subtype was also differentially enriched in H/ACA snoRNAs that are required for telomerase activity [59]. We identified several yRNA species in the LAR subtype that may be hormone dependent. There is increasing evidence that anti-androgen targeted therapies are efficacious in this subtype [60]. yRNA fragments have recently been found in the extracellular space of cultured breast cancer cells [61]. The ability to detect yRNA fragments in serum from breast cancer patients [62] may provide an opportunity for a minimally invasive way to serially monitor this TNBC subtype during treatment, similar to PSA for prostate cancer patients. We also found the 7SL snRNA component of the signal recognition complex enriched in the M subtype, suggesting that this subtype may be more dependent on processing secreted proteins. This finding is consistent with the enrichment in mRNAs encoding secreted growth factor and developmental signaling proteins. The diversity of small non-coding RNAs present in TNBC subtypes reflects the complexity of the disease and the variety of mechanisms to regulate tumor suppressor and oncogenes.

## Figures and Tables

**Table 1:** Summary of differentially expressed sRNAs between TNBC subtypes.

Comparison	miRNA	isomiR	tRNA	snRNA	snoRNA	yRNA	7SK	7SL	Total
BL1 vs. BL2	29	33	1	1	6	0	1	0	71
LAR vs. BL2	82	74	0	9	2	0	0	0	167
LAR vs. BL1	49	40	0	9	10	2	1	0	111
M vs. BL2	26	16	0	2	6	0	0	0	50
M vs. BL1	71	84	2	3	15	0	4	2	181
M vs. LAR	105	105	0	5	19	5	0	2	241
Total	362	352	3	29	58	7	6	4	821

**Table 2:** Summary of sRNAs unique to TNBC subtypes.

Comparison	miRNA	isomiR	tRNA	snRNA	snoRNA	yRNA	7SK	7SL	Total
BL1 vs. others	18	0	2	2	8	0	2	0	32
BL2 vs. others	6	0	0	0	0	0	0	0	6
M vs. others	41	0	0	2	3	0	0	2	48
LAR vs. others	82	0	0	10	6	6	0	0	104

**Table 3:** miRNA gene targets altered by TNBC subtype.

Subtype	Gene Class	Decreased miRNA Target	Increased miRNA
BL1	Oncogene	H3F3A	HRAS, MDM4
	Tumor Suppressor	DNMT3A	CDH1, BCOR, BAP1, FBXW7
BL2	Oncogene	EIF4A2, PPP2R1A	
	Tumor Suppressor	FBXW7	
M	Oncogene	MDM4, HRAS	CDK4, EIF4A2, FGFR1, H3F3A, MDM4, MYB, MYCL1, NRAS,
	Tumor Suppressor	FBXW7, CDH1	NOTCH2, SMAD2
LAR	Oncogene	EIF4A2, HF3F3A, MDM2, MDM4, MYCL1	CDK4, EIF4A2, ETV1, FGFR1, IDH2, MDM2, MDM4
	Tumor Suppressor	GATA3, VHL	FBXW7, NOTCH2

## Supplementary Materials

The following are available online at www.mdpi.com/2073-4425/9/1/29/s1. Figure S1: Unsupervised hierarchical clustering of TNBC cell lines by sRNA species. Figure S2: Correlation network for tumor suppressors and small non-coding RNAs in TNBC cell lines. Figure S3: Correlation network for oncogenes and small non-coding RNAs in TNBC cell lines. Table S1: The 26 TNBC cell line and their subtypes. Table S2: Detected sRNA and their read counts. Table S3: Unique sRNAs pertaining to each TNBC subtype. Table S4: Pathway analysis results. Table S5: List of oncogenes and tumor suppressor genes used for co-expression.
