# Supplementary figures and images for: The Landscape of Small Non-Coding RNAs in Triple-Negative Breast Cancer
Source: Genes (Basel). 2018 Jan 10;9(1):29. doi: 10.3390/genes9010029 (PMC5793181; doi:10.3390/genes9010029)

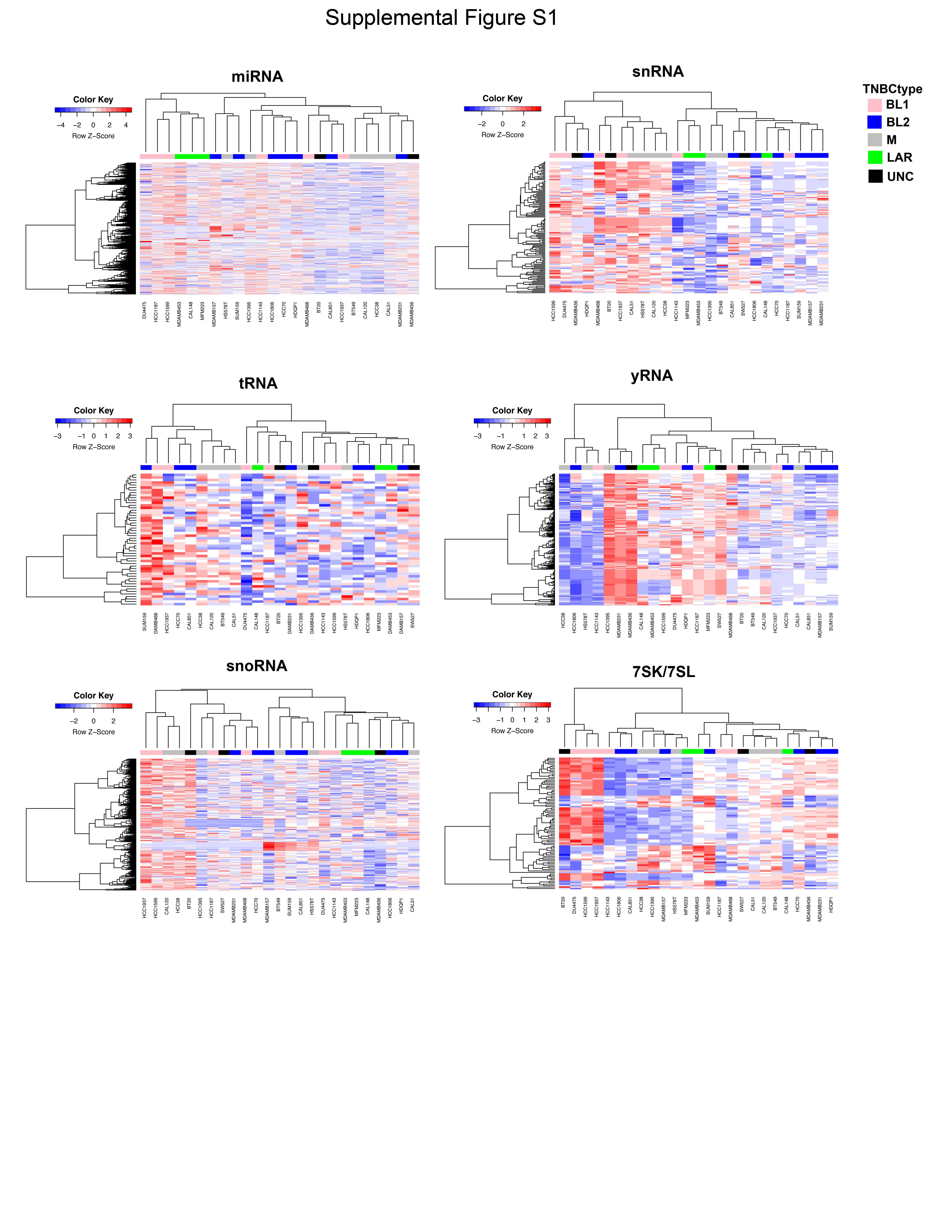

Supplement: Supplementary file 1 [file genes-09-00029-s001.zip › Supplemental_Figure_S1 unsupervise.jpg]

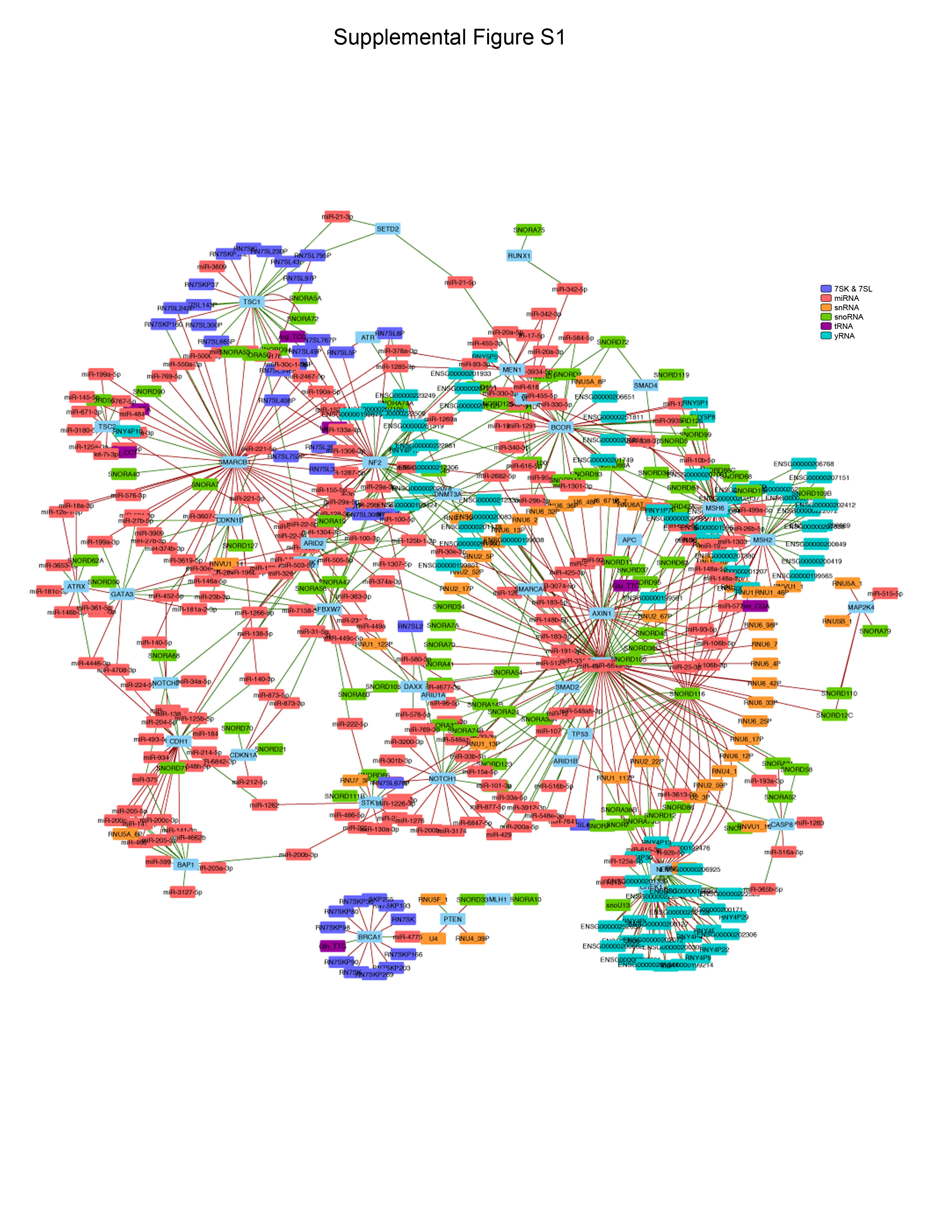

Supplement: Supplementary file 1 [file genes-09-00029-s001.zip › Supplemental_Figure_S2_TSG.jpg]

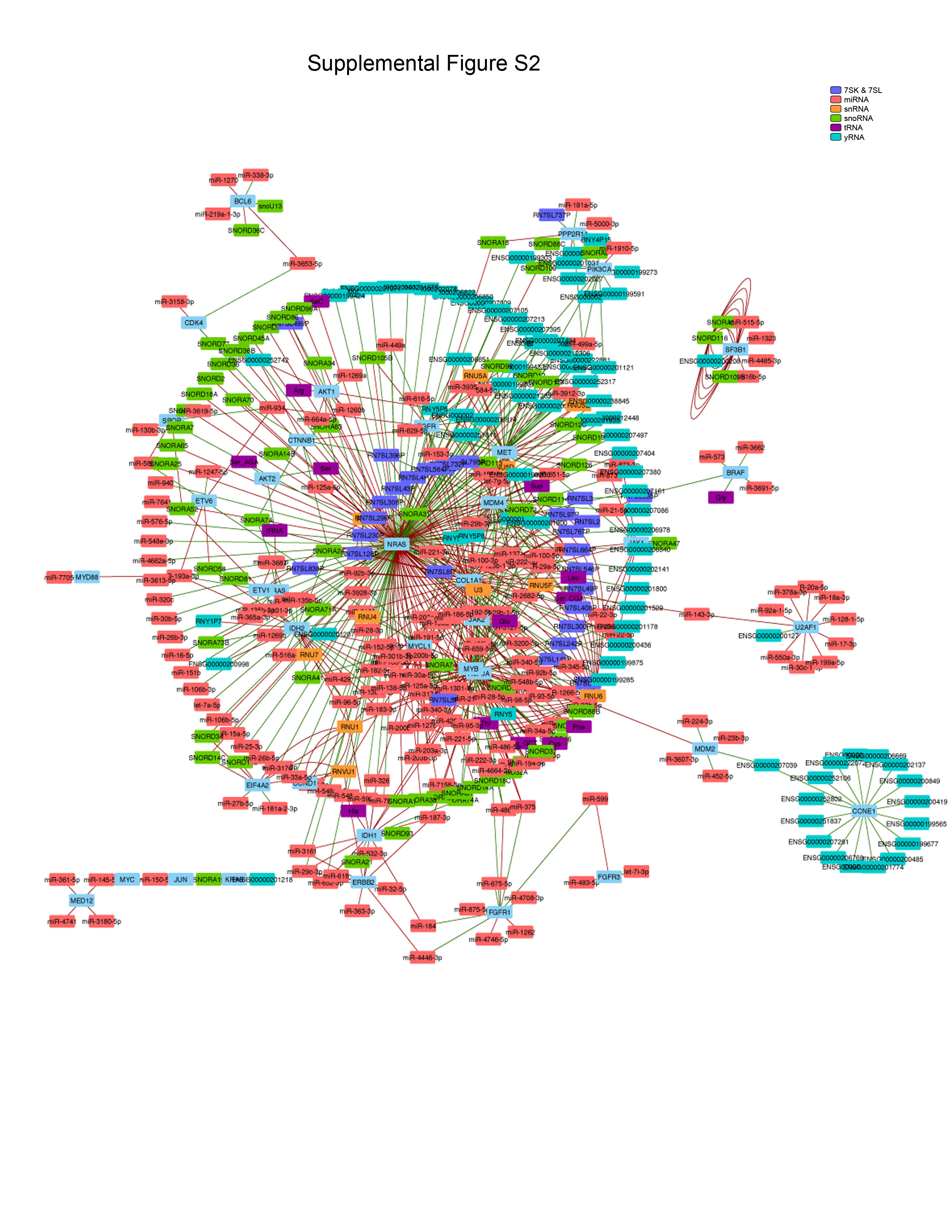

Supplement: Supplementary file 1 [file genes-09-00029-s001.zip › Supplemental_Figure_S3_OG.jpg]
